# Exploring Single-Cell Exposomics by Mass Spectrometry

**DOI:** 10.1021/acs.est.3c04524

**Published:** 2023-08-10

**Authors:** Peng Gao

**Affiliations:** † Department of Environmental and Occupational Health and Department of Civil and Environmental Engineering, University of Pittsburgh, Pittsburgh, Pennsylvania 15261, United States; ‡ UPMC Hillman Cancer Center, Pittsburgh, Pennsylvania 15232, United States

**Keywords:** single-cell exposomics, advanced mass spectrometry, cell-type-specific exposomics, spatial exposomics, precision environmental health

## Abstract

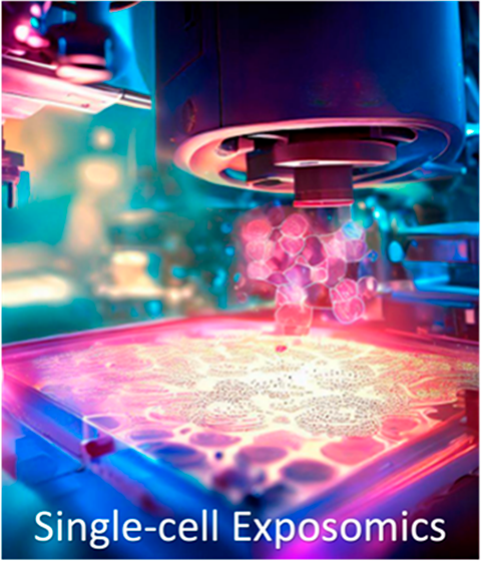

Single-cell exposomics, a revolutionary approach that investigates cell–environment interactions at cellular and subcellular levels, stands distinct from conventional bulk exposomics. Leveraging advancements in mass spectrometry, it provides a detailed perspective on cellular dynamics, interactions, and responses to environmental stimuli and their impacts on human health. This work delves into this innovative realm, highlighting the nuanced interplay between environmental stressors and biological responses at cellular and subcellular levels. The application of spatial mass spectrometry in single-cell exposomics is discussed, revealing the intricate spatial organization and molecular composition within individual cells. Cell-type-specific exposomics, shedding light on distinct susceptibilities and adaptive strategies of various cell types to environmental exposures, is also examined. The Perspective further emphasizes the integration with molecular and cellular biology approaches to validate hypotheses derived from single-cell exposomics in a comprehensive biological context. Looking toward the future, we anticipate continued technological advancements and convergence with other -omics approaches and discuss implications for environmental health research, disease progression studies, and precision medicine. The final emphasis is on the need for robust computational tools and interdisciplinary collaboration to fully leverage the potential of single-cell exposomics, acknowledging the complexities inherent to this paradigm.

## Abstract 2

Single-cell exposomics, powered by advanced mass spectrometry, reveals cellular responses to environmental stressors, having significant implications for disease studies and precision environmental health.

## 1 Introduction

The field of health sciences has seen a significant evolution over the past two decades in our understanding of the impact of environmental exposure on human health. This has largely been propelled by the concept of the exposome and the rapidly transforming field of exposomics, the systematic study of the exposome.^1^ As a burgeoning discipline, exposomics is accelerating research in environmental health sciences, unraveling the exposure factors contributing to human phenotype and disease susceptibilities.^2^ On the contrary, the integrations of single-cell analysis and various cutting-edge mass spectrometry (MS) methods offer unprecedented opportunities to unravel the complexity of environmental exposures at an individual cell level. The concept of single-cell exposomics has emerged from the need to understand how various exposures independently affect each cell, subsequently influencing its function and potential for disease development.^3^

Historically, exposomics analyses have been performed on bulk tissues or biospecimens such as the kidneys, liver, blood, and urine. However, this approach overlooks the unique characteristics and responses of individual cells.^1^ Considering the heterogeneity of cellular responses, the traditional bulk level analysis can mask significant variations and potentially lead to misleading interpretations.^4^ With the advent of single-cell technologies, these boundaries have been pushed, enabling exploration of the heterogeneity of cellular responses to environmental exposures. For instance, while environmental exposure may affect only a small proportion of cells within an organism, it can still instigate significant health changes that might be overlooked when evaluated at the population level.^5^

Single-cell exposomics offers significant potential because it provides insights into how individual cells respond to environmental stimuli, aiding in the understanding of the initiation, progression, or prevention of diseases. The cells’ interactions with their environment form the basis for understanding the organism’s overall health and disease state.^6^ Observing how each cell responds to environmental stimuli helps us uncover the interplay of genetic and environmental factors that lead to varying disease susceptibilities and biological responses among individuals. Therefore, this underlines the emerging necessity for a single-cell level analysis in the context of exposomics. However, a comprehensive and in-depth exploration of disease mechanisms requires the integration of other single-cell methods such as genomics, proteomics, and metabolomics as well as experimental validations. Consequently, while single-cell exposomics plays a critical role in gaining mechanistic insights into diseases, it is part of a broader approach that could potentially lead to improved prevention strategies and personalized medicine.

Examining cellular exposures enhances our understanding of how environmental exposures affect human health at the cellular level. For instance, investigating spatial aspects of cellular exposure, including the interactions between cells and their microenvironments, can yield valuable insights into the complex interplay of factors contributing to health outcomes. Essentially, single-cell exposomics serves as a window into the intricacies of cellular dynamics, offering a high-resolution image of our interaction with the environment.^7^ In addition, by exploring the specific responses of different cell types to environmental exposures, we can gain valuable insights into their distinct susceptibilities and defense mechanisms. This focused investigation enables us to understand how cells react uniquely to various exposures, uncovering their vulnerabilities and adaptive strategies. Such knowledge paves the way for targeted interventions and enhances our understanding of complex biological systems, empowering us to mitigate the adverse effects of environmental exposures on specific cell types and organelles.^8^ Leveraging the power of high-resolution MS, single-cell exposomics provides an exceptional level of detail, akin to that of single-cell metabolomics. As a nontargeted and spatial approach, MS can identify a wide range of compounds, making it an ideal tool for spatial exposome characterization.^9^ Overall, the success of MS in single-cell exposomics is attributed to its high sensitivity, specificity, and ability to measure a large variety of small biomolecules simultaneously.

This Perspective aims to discuss the latest developments in this sphere, emphasizing the potential of single-cell exposomics to deepen our understanding of cellular responses to chemical exposures and their impact on human health. Ultimately, single-cell exposomics propels us into the uncharted territory of understanding the intricate environmental interactions occurring at the level of individual cells, marking an exciting advancement in the field of environmental health sciences.

## 2 Spatial and High-Resolution Mass Spectrometry

Recent studies of small biomolecules within single cells have primarily employed four prominent methodologies: fluorescence-based detection, fluorescence biosensors, fluorescence resonance energy transfer biosensors, and MS. Among these, three fluorescence-based methods, which utilize microscopy to detect cellular molecules by applying fluorescent tags to targeted molecules, have been frequently chosen. However, these techniques have shown limitations within single-cell exposomics, as they have been observed to alter metabolite and xenobiotic activity due to their invasive nature. A currently favored workaround involves the use of fluorescent proteins that act as metabolite sensors, fluorescing upon binding with a molecule of interest.^10^

Nevertheless, MS has gained prominence as a preferred technique for single-cell analyses. The advantages are notable. It obviates the need to develop fluorescent proteins for each molecule of interest and can detect small biomolecules within the femtomole range.^11^ Taking cues from successful strategies in proteomics and metabolomics, the integration of MS with separation techniques like capillary electrophoresis or high-performance liquid chromatography has further facilitated biomolecule separation and annotation.^12^ Moreover, combining capillary microsampling with MS and ion mobility separation enhances molecular coverage and ion separation for MS single-cell analyses.^13^ This fusion of chromatography and spectrometry could significantly improve the sensitivity and comprehensiveness of exposure detection and characterization in single-cell exposomics.^14^

MS has long served as an essential tool in biological analysis, playing an invaluable role in characterizing complex molecular systems. It allows measurement of the mass-to-charge ratio of ions, which can be used to determine the elemental composition, structure, and quantity of various molecules. As such, MS’s versatility, sensitivity, and precision have cemented its position in biomonitoring.^15^ Recently, there has been a paradigm shift in the application of MS from bulk sample studies to examination of individual cells. This transformation has been spurred by advancements in spatial and high-resolution MS, which empower researchers to probe the spatial organization of biomolecules within single cells, providing an unprecedented view of the cellular landscape.^16^ Spatial MS, or imaging MS, enables the direct analysis of biological samples, creating detailed molecular distribution maps within cells and tissues. This spatially resolved data allow us to determine which molecules are present and their specific locations within the cell.^17^ This spatial awareness can unveil unique cellular substructures and variations essential for understanding cell functions and responses to environmental exposures. In contrast, high-resolution MS offers a detailed view of the molecular composition of samples. It can distinguish between ions with closely related mass-to-charge ratios, enabling the identification and quantification of thousands of molecules in single cells.^18^ This high level of detail is critical in exposome studies, as it allows the detection of subtle changes in cellular composition in response to environmental exposures.

Substantial advancements in MS technology have set the stage for single-cell exposome analysis. Developments in sample preparation techniques, such as microscale sample handling and nanoscale liquid chromatography, permit the isolation and analysis of individual cells.^19^ Similarly, advancements in ionization techniques, such as nanoelectrospray ionization, enable the generation of ions from minute amounts of sample, facilitating MS analysis at the single-cell level.^20^ When combined with progress in data analysis and machine learning techniques to manage vast amounts of generated data, these breakthroughs position us at the leading edge of single-cell exposomics. This progress allows for a comprehensive exploration of cellular interactions with the environment at a level of detail previously unattainable.

## 3 Spatial Single-Cell Exposomics

The intersection of spatial MS with single-cell analysis has forged a new path within exposomics, giving rise to the burgeoning field of spatial single-cell exposomics. This cutting-edge domain harnesses the capabilities of spatially resolved MS to explore how individual cells respond to and adapt to environmental exposures. With the dual power of high-resolution and spatial MS, we can construct detailed distribution maps of environmental exposure within individual cells. This approach illuminates not only the presence of specific compounds but also their locations within cells or tissues, offering insights into how these interact with cellular components and shape cellular function.^21^

Spatial single-cell exposomics offers valuable insights by detecting environmental contaminants and their biotransformation products and by mapping their distribution patterns within the cell. The connection between spatial organization and biological function at the single-cell level is pivotal. Cellular functions are intrinsically linked to spatial contexts: the location of molecules can significantly influence their roles and interactions.^22^ Thus, in single-cell exposomics, discerning the spatial distribution of molecules can illuminate the intricate mechanisms of cellular responses to environmental stimuli. For instance, the entry of environmental toxins into a cell can disrupt the spatial organization of proteins and lipids. This can alter vital cellular processes, leading to phenotypic changes such as modified cell behavior or an altered response to signals. In extreme cases, this could even steer a cell toward a diseased state.^23^ Therefore, spatial single-cell exposomics offers critical insights into cellular responses to environmental pollutants, aiding our understanding of disease onset and progression. However, while MS-based single-cell exposomics can characterize the spatial distribution of xenobiotics, a comprehensive understanding of how cells interact with and adapt to their environment requires integrated multiomics approaches. For instance, a comprehensive understanding of host responses to environmental exposures would ideally be achieved by incorporating data from both single-cell exposomics and single-cell metabolomics.

**Figure 1:**
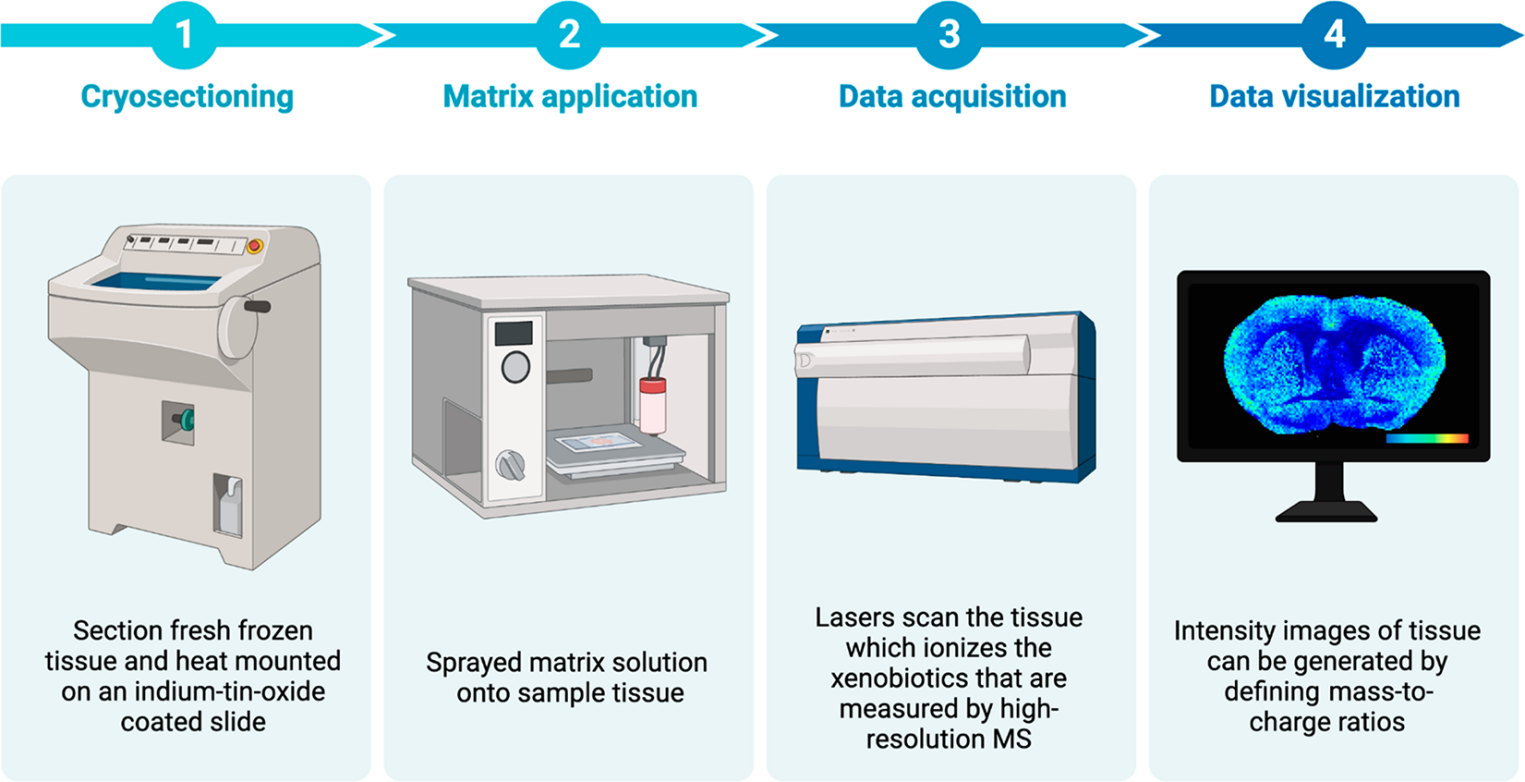
Illustrative workflow of profiling a spatial single-cell exposome. Created with BioRender.com.

A typical spatial single-cell exposomics workflow by matrix-assisted laser desorption/ionization coupled with MS first commences with cryosectioning, a technique used to prepare thin, frozen sections of the biological sample for spatial analysis. Once the cryosections are prepared, a matrix solution will be applied. This matrix assists in the desorption and ionization of the compounds in the sample for subsequent MS analysis. Data for the prepared sample are then acquired by spatial MS, which measures the mass-to-charge ratio of the ions in the sample. Typically, matrix-assisted laser desorption/ionization and advanced methodologies, such as nanoscale secondary ion mass spectrometry, can enable detailed mapping within individual cells and tissue sections. These techniques detect a broad spectrum of chemicals with high-resolution MS instruments capable of identifying numerous molecules in one run, covering diverse chemical classes. The sensitivity of modern MS technologies allows for the detection of compounds within a wide concentration range. As such, these techniques enable the visualization of the spatial distribution of xenobiotics within individual cells, providing insight into the molecular composition and organization within cells. Finally, the MS data are processed and visualized using specialized software or pipelines (Figure 1). This visualization can take the form of heat maps or other graphical representations, providing a spatially resolved view of the xenobiotics present in the sample. This data visualization step allows researchers to study the intricate interplay between environmental exposures and cellular responses.^24^

Spatial single-cell exposomics can be harnessed to address intricate biological questions across numerous applications. For example, one could generate high-resolution spatial exposome and metabolome maps of single cells to comprehend the metabolic heterogeneity and adaptive strategies of yeast cells in response to environmental stimuli.^25^ This approach could highlight the immense potential of spatial single-cell exposomics in delivering a nuanced picture of cellular responses and adaptations right down to subcellular regions. The applications can be also extended into biomedical fields like neurobiology, where single-neuron spatial metabolomics can be used to map neurotransmitter distributions in identified neurons.^26^ Spatial exposomics can then map neurotoxin distributions, illustrating how incorporating the spatial dimension could enhance our understanding of single-cell neuronal function and complex responses to xenobiotics. In addition, the tool can be set to revolutionize oncology by providing a detailed understanding of the tumor microenvironment. By delineating the spatial distribution of environmental carcinogens and their biotransformation products at the single-cell level within precancerous cells, this tool allows us to delve into the underpinnings of carcinogenic mechanisms.^27^ It explains the perplexing observation of why certain cells evolve into cancer cells while others do not, despite experiencing similar environmental exposures. This heterogeneity in carcinogen distribution could underlie variations in genetic alterations, leading to disparities in carcinogenesis and cellular response, thereby unlocking new possibilities for early detection strategies and personalized prevention.^28^ Moreover, spatial single-cell exposomics can be coupled with other single-cell technologies such as single-cell RNA sequencing (scRNA-seq) or single-cell ATAC sequencing, allowing for simultaneous examination of the transcriptome or accessible chromatin within the same cell. This integrated approach can elucidate connections between the exposome and the cellular phenotype, enabling a deeper understanding of the impact of environmental exposures on cellular function and disease pathogenesis.^6^

In conclusion, spatial single-cell exposomics not only is an innovative analytical approach but also leads to a new dimension of understanding the cellular exposome. With continued refinement and advancements, this holds tremendous potential for reshaping our understanding of cellular function and disease processes, offering a new lens through which we can explore the intricate tapestry of cellular interactions with the microenvironment.

## 4 Single-Cell-Type Exposomics

Like other -omics technologies, the field of exposomics has traditionally faced a key challenge, the lack of resolution at the cellular level. This issue becomes especially prominent when considering the heterogeneity existing not only among different cell types but also within a single cell type.^29^ Single-cell-type exposomics, a newly emerging field, is poised to bridge this gap, enabling researchers to explore how varying cell types respond to identical environmental exposures. By leveraging this approach, we can investigate the range of environmental exposures encountered by different cell types and comprehend their impacts on cellular function and health outcomes. This novel perspective could usher in a new era in precision environmental health, fostering the development of highly targeted intervention and prevention strategies.^30^

The principle of single-cell-type exposomics underscores the fact that cellular responses to environmental exposure are far from uniform. By offering unique insights into the differential susceptibility and adaptive strategies of various cell types, it highlights the rich tapestry of cellular diversity.^31^ This focus on individual cell types allows researchers to decode the intricate complexity and diversity within tissues, catalyzing the emergence of personalized, cell-specific preventive approaches. Such cell-type-specific exposomics can illuminate why certain cells might be more vulnerable to specific environmental stressors. For instance, specific neurons can demonstrate an increased vulnerability to certain neurotoxins, a phenomenon attributed to the unique characteristics that they possess. This heightened susceptibility is often rooted in the exclusive set of receptors they express, with certain neurotoxins having a higher affinity for these specific receptors. Simultaneously, the distinct metabolic profiles of these neurons, encompassing energy production, protein synthesis, and detoxification pathways, can further contribute to their sensitivity.^32^ Thus, single-cell-type exposomics plays a pivotal role in unraveling cell-specific mechanisms of disease pathogenesis and progression, which could be masked in bulk tissue analysis.

Despite its immense potential, cell-type-specific exposome analysis comes with its own set of technical challenges. One important issue is the hurdle of chemical annotation when using high-resolution MS, which is a common challenge in exposomics research. Accurately identifying and quantifying the wide array of environmental chemicals in a single cell can be a daunting task due to the complexity and diversity of the exposome. Current limitations in database completeness and spectral libraries also add to the difficulty of precise chemical annotation. This aspect needs to be further developed and improved for more accurate and comprehensive chemical annotation in single-cell exposomics. Other considerations include the potential limitations introduced by preselecting cell types for analysis, which may limit the discovery of new cell types with different exposure or metabolite distributions. Therefore, it can be more beneficial to conduct single-cell experiments on tissue with an unknown number of cell types first, followed by data analysis to identify the cell types. The evolution of advanced techniques, such as fluorescence-activated cell sorting and laser-capture microdissection, has now made this precision isolation feasible.^33^ Coupled with breakthroughs in microfluidics technologies and droplet-based platforms, single-cell isolation has become more efficient and less invasive.^34^ After cell isolation and type identification based on data frmo single-cell experiments, high-resolution MS can be employed for single-cell exposomics, akin to bulk analysis.^35^ To provide insights at the cellular and subcellular levels, high-resolution MS like Orbitrap and time-of-flight MS can be employed due to their broad chemical detection capabilities and sensitivity. These advanced MS methods can identify a variety of chemicals, demonstrating wide-ranging detection limits. This workflow enhances the potential to enrich or purify specific sample types, revealing the unique responses of each cell type to varying environmental exposures and ultimately facilitating a more detailed and comprehensive understanding of cellular responses within the realm of exposomics.

**Figure 2:**
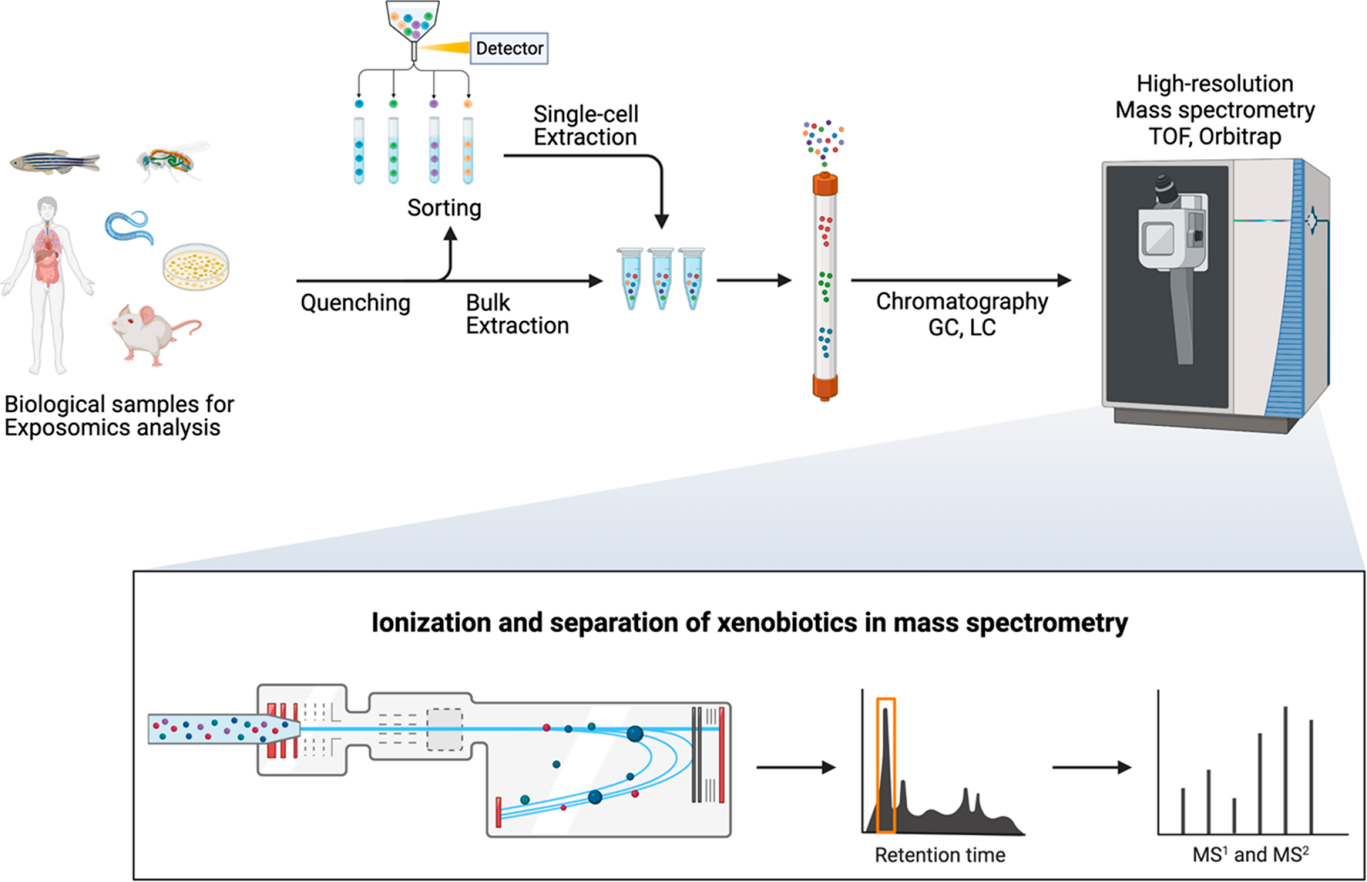
Illustrative workflow of profiling bulk and single-cell-type exposomes.^36^ Abbreviations: GC, gas chromatography; LC, liquid chromatography; TOF, time-of-flight; MS, sass spectra. Created with BioRender.com.

A typical workflow of single-cell-type exposomics begins with the selection of biological samples that can be obtained from human subjects or model organisms, depending on the specific research question and objectives. After suitable biological samples are chosen, the next step is quenching, which involves introducing a specific solution into the biological system to rapidly halt all enzymatic activity, preserving the current state of xenobiotics. This process is fundamental to ensuring an accurate snapshot of the system at the time of sampling. Once quenched, the samples are kept at low temperatures to prevent any changes. Next, the process moves to cell sorting or other types of isolation. This step is crucial for separating the desired cell type from the rest of the sample, allowing for a more accurate analysis. Given the quenching step, biotransformable chemicals should remain inactive during cell sorting. However, it is important to consider that some cell isolation techniques might disrupt the native state of cells and affect the accuracy of single-cell exposomics experiments. Therefore, it is crucial to adopt techniques that allow for less invasive and more efficient cell isolation, keeping the cells’ native state intact as much as possible. Following cell isolation, the xenobiotics are then extracted from the cells. Different methods can be used for extraction, depending on the nature of the xenobiotics and the type of cells. The extracted xenobiotics are then analyzed through various chromatographies coupled with high-resolution MS. Chromatography allows the separation of the mixture of xenobiotics based on their physicochemical properties. Subsequently, high-resolution MS provides an accurate measurement of the mass-to-charge ratios of the xenobiotics, enabling the annotation and/or identification of the compounds present in the sample (Figure 2).^36^ The complex data obtained from MS will be processed and interpreted by using advanced bioinformatics tools and statistical methods. The aim is to uncover meaningful insights into the exposure of different cell types to xenobiotics, their response mechanisms, and potential health implications.

Single-cell-type exposomics offers several applications with the potential to drive significant discoveries. For instance, one could conduct single-cell exposome analysis on different lung cell types to scrutinize their differential responses to particulate matter exposures.^37^ The expected findings could demonstrate that distinct cell types exhibit varying adaptive metabolic responses, underlining the importance of cell-specific analyses in deciphering complex pathophysiological processes. Additionally, single-cell-type exposomics can be employed to examine the responses of hepatocytes, vital cells for detoxification, to various environmental toxins and carcinogens.^38^ Such studies could expose a high degree of biotransformation heterogeneity among hepatocytes when dealing with xenobiotics, emphasizing the necessity to factor in cell-type specificity when assessing cellular responses to environmental stressors. Furthermore, the integration of single-cell-type exposomics with other single-cell technologies such as scRNA-seq or single-cell proteomics could provide a multiomics perspective on the cellular response to environmental exposures. For example, by coupling xenobiotic profiling with scRNA-seq, one could examine not only the types of xenobiotics to which different cell types are exposed but also the changes in gene expression that occur in response to these exposures. This integrated approach could lead to the identification of novel molecular pathways involved in disease onset and progression caused by xenobiotic exposures.^6^

In summary, single-cell-type exposomics represents a potent tool for dissecting cell-specific responses to environmental exposures. Despite the technical obstacles, advancements in single-cell isolation technologies and high-resolution analytical tools are projected to further extend the frontiers of this field, illuminating cell-specific disease mechanisms and potential prevention strategies in ways previously unimagined.

## 5 Deepening Integration with Molecular and Cellular Biology

Exposomics reaches beyond the realm of environmental exposures, incorporating the biological responses at the individual cellular level to these exposures, thereby linking to health outcomes.^1^ As such, single-cell exposomics necessitates a robust understanding of molecular and cellular biology, as it is rooted in the principles of these fields. This specialized approach deciphers the complex interactions between environmental factors and cellular components, such as DNA, RNA, proteins, and metabolites, which form the basis of cellular responses to environmental exposures.^39^ Therefore, an intimate relationship with molecular and cellular biology is pivotal not only for the successful implementation of single-cell exposomics but also for the significant interpretation of its data. By providing the requisite biological context, these fields facilitate the translation of exposome data into a comprehensive understanding of the ways environmental factors impact cellular function and health.^23^ Additionally, single-cell exposomics holds a potential symbiosis with other single-cell techniques, such as single-cell DNA sequencing (scDNA-seq), scRNA-seq, and single-cell metabolomics. These techniques provide additional layers of information about the cell’s status, revealing how the exposome is translated into cellular function and health.

The complex interplay of cellular processes dictates the cellular responses to environmental exposures, whether they are adaptive, protective, or maladaptive.^40^ For instance, the effects of diverse environmental toxins on a cell could be modulated by the cell’s detoxification mechanisms, such as cytochrome P450 enzymes,^41^ which are regulated by gene expression, signaling pathways, and other cellular mechanisms.^42^ Therefore, a profound understanding of these cellular processes is crucial for interpreting the results of single-cell exposomics. Molecular biology also plays a pivotal role in exposomics, providing tools such as next-generation sequencing and CRISPR-Cas9 gene editing that can elucidate the molecular underpinnings of the observed cellular responses to environmental exposures.^43^ Researchers can employ these tools to study changes in gene expression or alterations in DNA methylation that occur in response to specific environmental stressors. By doing so, they can start to unravel the complex molecular pathways involved in cellular adaptation and the potential triggers for disease development.^44^ Beyond molecular biology tools, the integration of single-cell exposomics with techniques such as scDNA-seq, scRNA-seq, and single-cell metabolomics adds further depth to the analysis. For example, combining scRNA-seq with single-cell exposomics could help connect specific environmental exposures to changes in gene expression, shedding light on the molecular mechanisms of cellular responses to the environment. Similarly, single-cell metabolomics can complement single-cell exposomics by providing insights into metabolic changes in response to environmental exposures. This integration can result in a multiomics approach at the single-cell level, offering a more holistic view of cellular responses to environmental exposures.

**Figure 3:**
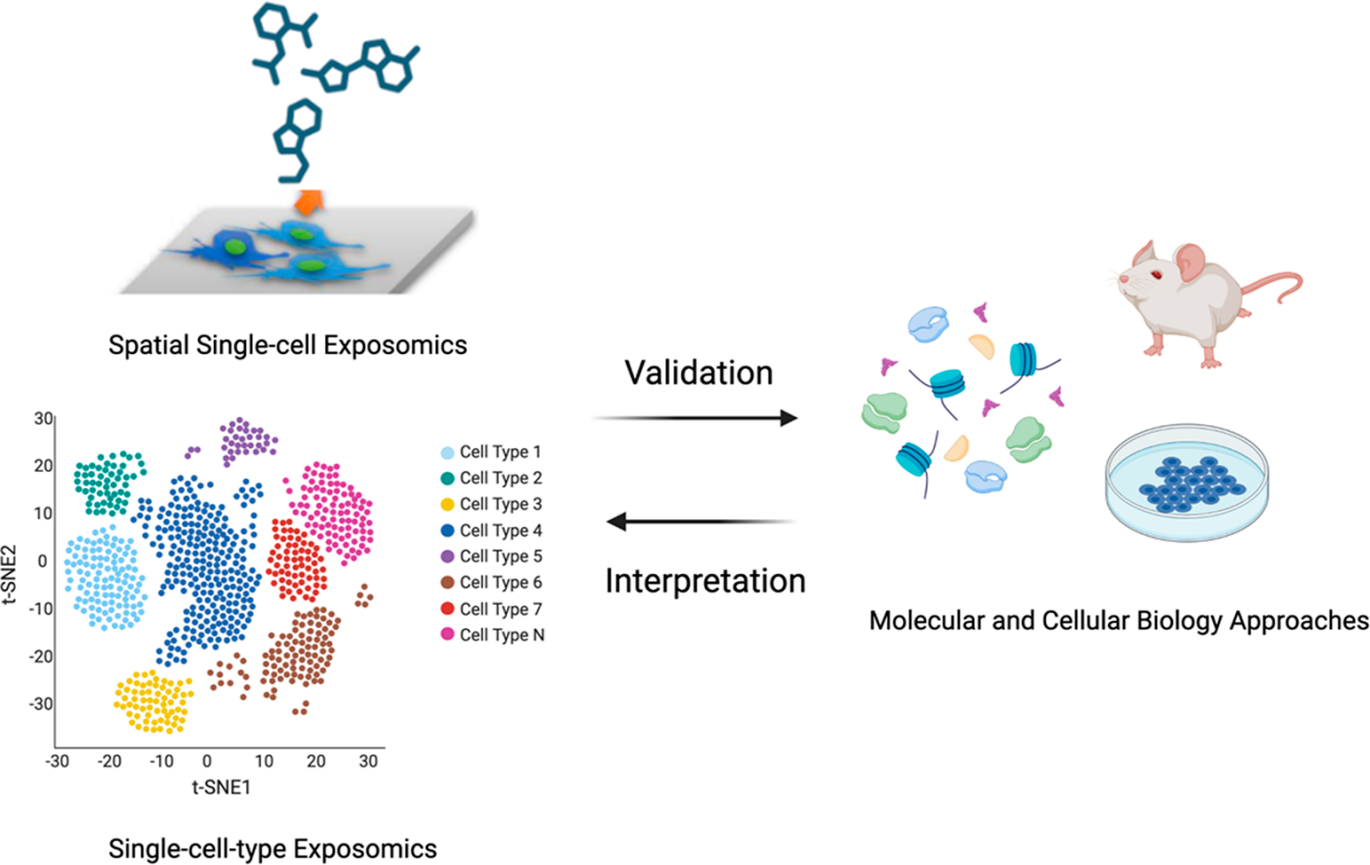
Integrating single-cell exposomics with molecular and cellular biology approaches to validate and interpret environmental and biomedical implications.^17^ Created with BioRender.com.

Single-cell exposomics, as an omics approach, generates extensive and detailed data regarding the environmental exposures experienced by individual cells. However, to validate the hypotheses generated from abundant data, it is critical to integrate insights from molecular and cellular biology. This is due to the fact that exposomics data, while comprehensive, often require validation and context to understand the biological significance of observed phenomena.^1^ Molecular and cellular biology approaches can provide this context, offering mechanistic insights into how specific exposures might impact cellular function and behavior. For example, if exposomics data suggest specific xenobiotics could affect a cellular process, *in vitro* and *in vivo* mechanistic studies could elucidate the exact molecular pathways involved, the specific cellular components impacted, and the broader implications for cell function.^45^ Thus, molecular and cellular biology approaches play an indispensable role in the interpretation, validation, and applicability of hypotheses generated by single-cell exposomics (Figure 3). However, such integration necessitates the application of sophisticated bioinformatic tools for multiomics data integration, interpretation, and visualization. Therefore, a strong alliance with bioinformatics and computational biology is crucial for single-cell exposomics, helping to harness the full potential of the data generated.

Integration of single-cell exposomics with molecular and cellular biology offers an opportunity to explore the mechanisms underlying cellular responses to environmental exposures. This approach can provide insights into how these exposures influence health and disease at the molecular level, leading to the development of novel biomarkers and prevention strategies. For example, single-cell exposomics can be combined with proteomics to investigate the protein binding effects of exposure to a specific environmental carcinogen on individual lung cells.^46^ This approach could reveal a diverse range of individual cellular responses, driven by complex molecular pathways, that would have been obscured in traditional bulk analysis. Similarly, single-cell exposomics can pair with transcriptomics to examine the impact of air pollution on brain cells.^47^ This integrative approach can uncover specific molecular pathways involved in the cellular response to air pollutants, offering new insights into how environmental toxins might contribute to neurodegenerative diseases.

In conclusion, the deepening of the integration of molecular and cellular biology with single-cell exposomics and other single-cell techniques is essential. By bridging the gap between these disciplines, we can truly appreciate the complex and heterogeneous cellular responses to environmental exposure and better predict the health consequences. With the continuing advancement in molecular and cellular biology techniques, we can anticipate a future in which single-cell exposomics will provide even more detailed, precise, and meaningful insights into our biological responses to the world around us.

## 6 Future Perspective

Undoubtedly, single-cell exposomics facilitated by advancements in MS holds significant potential to revolutionize our understanding of the intricate interplay between environmental exposures and human health. It provides a detailed exposome map of individual cells, elucidating the cellular heterogeneity in response to environmental stressors and revealing novel mechanisms of disease onset and progression. Understanding how cells adapt to environmental stressors at a single-cell level could provide insights into why certain cells become pathogenic, while others remain healthy, enabling early disease detection and prevention strategies.

Technical advancements are expected to drive significant progress in this burgeoning field, contributing to future environmental health research, disease progression studies, and precision medicine.^30^ Innovations that enhance the speed and accuracy of single-cell isolation, compound identification, and data processing will likely be key areas of development. These include high-throughput techniques, offering enhanced sensitivity for xenobiotics, reliable replicability, and the capacity for accurate xenobiotic identification and quantification. The MS advancements over the past decades have allowed for targeted, suspect, and nontargeted analyses of contaminants, delivering unprecedented chemical space coverage, precision, and accuracy.^48^ Given the enormous amount and high dimensionality of data generated from single-cell exposomics, the need for robust computational tools for effective analysis and interpretation is crucial. The evolution of MS techniques has been spurred by the availability and progression of big-data approaches, including data mining, chemometrics, bioinformatics, and machine learning, all powered by ever-increasing computational capabilities.^39^ Advanced algorithms assist in identifying patterns and trends in the data, enabling the prediction of cellular responses based on exposure profiles and translating the complex data into actionable insights. The marriage of bioinformatics/cheminformatics and machine learning with single-cell exposomics is a vital component of modern research, aiding navigation through the immense data landscape of individual cell responses to various environmental exposures.^2^

The potential integration of single-cell exposomics with other omics approaches such as genomics, epigenomics, transcriptomics, proteomics, and/or metabolomics is another promising direction. This convergence offers a holistic view of the cell’s response to environmental exposure, from exposure to cellular response, and the eventual health outcome, helping to unravel the complex biological networks and pathways that mediate these responses.^49^ An ideal study design for such an application would necessitate a thoughtful consideration of the sample size, which should adequately cover the diversity of cell types and the range of exposure conditions. Longitudinal sampling could be incorporated, providing a dynamic understanding of the cellular responses to environmental exposures over time. In terms of environmental health, single-cell exposomics will enhance our ability to conduct more precise risk assessments of environmental pollutants and identify health-threatening exposures. By understanding how different cell types react to various environmental toxins, we can identify novel cellular targets for environmental health prevention and intervention.^2^ Moreover, this approach aligns with the vision of precision medicine. By combining single-cell exposomics with individual genetic and phenotypic information, we can develop personalized exposure risk profiles, guiding interventions ranging from tailored environmental exposure reduction strategies to individualized drug treatments.^30,49^ However, single-cell exposomics is still a budding field with a set of challenges and limitations. Ensuring sensitivity, accuracy, confidence, and efficient data throughput of the utilized technologies is an ongoing task. The complexity and sheer volume of the data generated necessitate sophisticated computational methods, requiring collaborative efforts across biology, chemistry, environmental science, and computational science. Important ethical aspects, such as data privacy and clinical use of this technology, also demand attention. Furthermore, the challenges related to robustness, reproducibility, and interpretation of high-dimensional data cannot be ignored. Notwithstanding these obstacles, continuous advancements in high-throughput technologies, bioinformatics, and machine learning are paving the way forward, holding great promise for the future of single-cell exposomics.^50^

In conclusion, while there is much work to be done, the journey toward a comprehensive understanding of the exposome is undoubtedly an exciting one, filled with the promise of novel discoveries and innovations. Single-cell exposomics presents a new frontier in our quest to understand the intricate relationship between environmental exposures and human health. The insights gained from this endeavor will shape the future of health and medicine, bringing us closer to the vision of personalized and precision healthcare. As scientists continue to explore this field, we can anticipate a profound impact on our understanding of human health and disease from an environmental perspective.
